# Harnessing Evolution to Elucidate the Consequences of Symbiosis

**DOI:** 10.1371/journal.pbio.1002066

**Published:** 2015-02-10

**Authors:** Nicole M. Gerardo

**Affiliations:** Department of Biology, Emory University, Atlanta, Georgia, United States of America

## Abstract

Many organisms harbor microbial associates that have profound impacts on host traits. The phenotypic effect of symbionts on their hosts may include changes in development, reproduction, longevity, and defense against natural enemies. Determining the consequences of associating with a microbial symbiont requires experimental comparison of hosts with and without symbionts. Then, determining the mechanism by which symbionts alter these phenotypes can involve genomic, genetic, and evolutionary approaches; however, many host-associated symbionts are not amenable to genetic approaches that require cultivation of the microbe outside the host. In the current issue of *PLOS Biology*, Chrostek and Teixeira highlight an elegant approach to studying functional mechanisms of symbiont-conferred traits. They used directed experimental evolution to select for strains of *Wolbachia* wMelPop (a bacterial symbiont of fruit flies) that differed in copy number of a region of the genome suspected to underlie virulence. Copy number evolved rapidly when under selection, and wMelPop strains with more copies of the region shortened the lives of their *Drosophila* hosts more than symbionts with fewer copies. Interestingly, the wMelPop strains with more copies also increase host resistance to viruses compared to symbionts with fewer copies. Their study highlights the power of exploiting alternative approaches when elucidating the functional impacts of symbiotic associations.

## Abstract 2

Microbial symbionts and their interaction with their hosts are challenging to study. This Primer assesses a new study that solves the problem by using experimental evolution to link symbiont genotype to host phenotype. Read the Research Article.

Symbioses, long-term and physically close interactions between two or more species, are central to the ecology and evolution of many organisms. Though “Symbiosis” is more often used to define interactions that are presumed to be mutually beneficial to a host and its microbial partner, a broader definition including both parasitic and mutualistic interactions recognizes that the fitness effects of many symbioses are complex and often context dependent. Whether an association is beneficial can depend on ecological conditions, and mutation and other evolutionary processes can result in symbiont strains that differ in terms of costs and benefits to hosts (Fig. 1).

**Fig 1:**
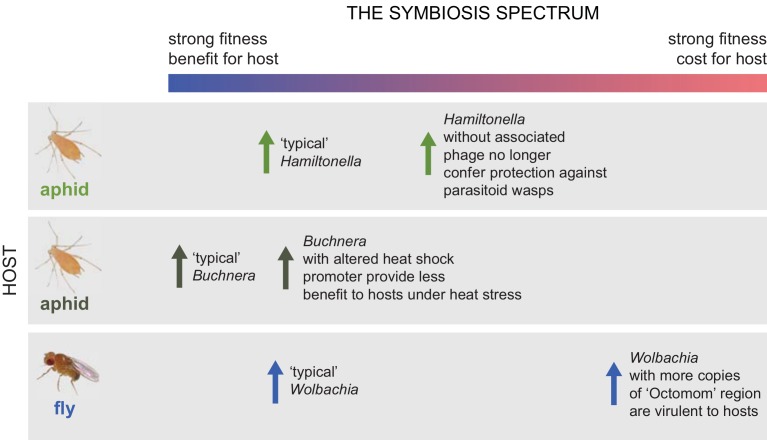
The symbiosis spectrum. The costs and benefits of symbiosis for hosts are not bimodal but span a continuum. The benefit to cost ratio is mediated both by environmental conditions and by the strain of symbiont. For example, the bacteria *Hamiltonella defensa* increases aphid resistance to parasitoid wasps. When *Hamiltonella* loses an associated bacteriophage, protection is lost. Also, in aphids, *Buchnera aphidicola* is a bacterial symbiont that provisions its hosts with critical nutritional resources. However, alterations of the heat shock promoter in *Buchnera* lessen the fitness benefit of symbiosis for the hosts under elevated temperatures. Amplification of a region of the *Wolbachia* genome known as Octomom causes the bacteria to shorten the lifespan of its *Drosophila* fly hosts.

Elucidating the effects of host-associated microbes includes, when possible, experiments designed to assay host phenotypes when they do and do not have a particular symbiont of interest (Fig. 2). In systems in which hosts acquire symbionts from the environment, hosts can be reared in sterile conditions to prevent acquisition [1]. If symbionts are passed internally from mother to offspring, antibiotic treatments can sometimes be utilized to obtain lineages of hosts without symbionts [2]. The impacts of symbiont presence on survival, development, reproduction, and defense can be quantified, with the caveat that these impacts may be quite different under alternative environmental conditions. While such experiments are sometimes more tractable in systems with simple microbial consortia, the same experimental processes can be utilized in systems with more complex microbial communities [3,4].

**Fig 2:**
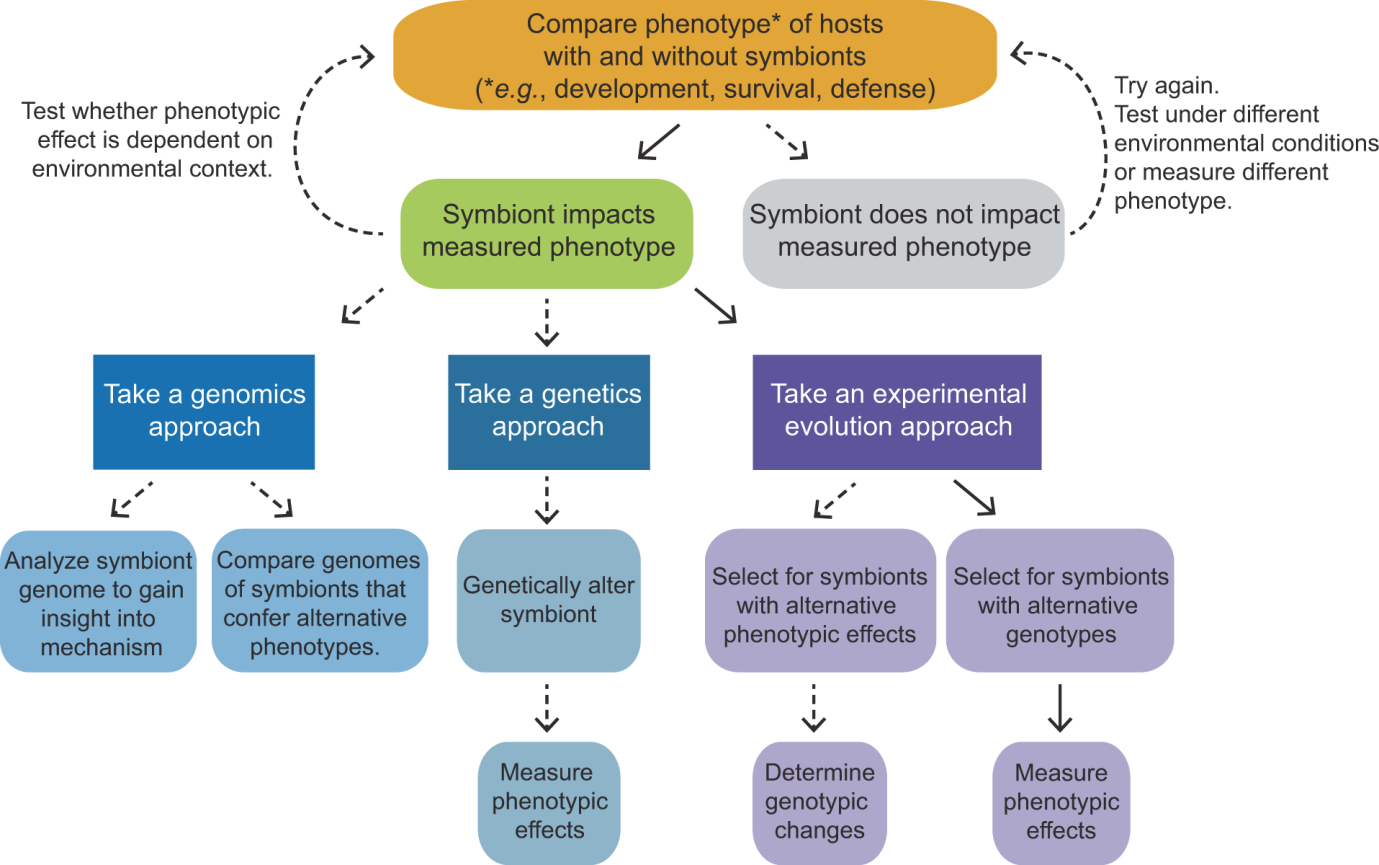
Approaches to functionally characterize symbiont effects. The first step in functionally characterizing the phenotypic impacts of a symbiont on its host is to measure phenotypes of hosts with and without symbionts. Any effects need to be considered in the light of how they are modified by environmental conditions. Understanding the mechanisms underlying symbiont alteration of host phenotype can involve, and often combines, genomic, genetic, and evolutionary approaches. Solid arrows indicate the path leading to results highlighted in Chrostek and Teixeira’s investigation of *Wolbachia* virulence in this issue of *PLoS Biology*.

Once a fitness effect of symbiosis is ascertained, determining the mechanistic basis of this effect can be challenging. A genomics approach sometimes provides informative insight into microbial function. Sequencing of many insect-associated symbionts, for example, has confirmed the presence of genes necessary for amino acid and vitamin synthesis [5–8]. These genomic revelations, in some cases, can be linked to phenotypic effects of symbiosis for the hosts. For example, aphids reared in the absence of their obligate symbiotic bacteria, *Buchnera aphidicola*, can survive when provisioned with supplemental amino acids but cannot survive without supplementation, suggesting that *Buchnera’*s provisioning of amino acids is critical for host survival [9,10]. The *Buchnera* genome contains many of the genes necessary for amino acid synthesis [5].

Linking genotype to phenotype, however, can be complicated. Experiments are necessary to functionally test the insights garnered from genome sequencing. For example, just because a symbiont has genes necessary for synthesis of a particular nutrient does not mean that the nutrient is being provisioned to its host. Furthermore, in many systems we do not know what genetic mechanisms are most likely to influence a symbiont-conferred phenotype. For example, if hosts associated with a given microbe have lower fitness than those without the microbe, what mechanism mediates this phenotype? Is it producing a toxin? Is it using too many host resources? In these cases, a single genome provides even less insight.

Comparative genomics can be another approach. This requires collection of hosts with alternative symbiont strains and then testing these strains in a common host background to demonstrate that they have different phenotypic effects. Symbiont genomes can then be sequenced and compared to identify differences. This approach was utilized to compare genomes of strains of the aphid bacterial symbiont *Regiella insecticola* that confer different levels of resistance to parasitoid wasps [11]; the protective and nonprotective *Regiella* genome differed in many respects. Comparing the genomes of *Wolbachia* strains with differential impacts on fly host fitness [12,13] revealed fewer differences, though none involved a gene with a function known to impact host fitness. Comparative genomics rarely uncovers a holy grail as the genomes of symbiont strains with alternative phenotypic effects rarely differ at a single locus of known function.

Another approach, which is at the heart of studies of microbial pathogens, is to use genetic tools to manipulate symbionts at candidate loci (or randomly through mutagenesis) and compare the phenotypic effects of genetically-manipulated and unmanipulated symbionts. Indeed, this approach has provided insights into genes underlying traits of both pathogenic [14] and beneficial [15,16] microbes. There is one challenge. Many host-associated symbionts are not cultivable outside of their hosts, which precludes utilization of most traditional genetic techniques used to modify microbial genomes.

An alternative approach to studying symbiont function leverages evolution. Occasionally, lineages that once conferred some phenotypic effect, when tested later, no longer do. If symbiont samples were saved along the way, researchers can then determine what in the genome changed. For example, pea aphids (*Acyrthosiphon pisum*) harboring the bacteria *Hamiltonella defensa* are more resistant to parasitoid wasps than those without the bacteria [17,18]. Toxin-encoding genes identified in the genome of a *Hamiltonella*-associated bacteriophage were hypothesized to be central to this defense [18,19]. However, confirmation of the bacteriophage’s role required comparing the insects’ resistance to wasps when they harbored the same *Hamiltonella* with and without the phage. No *Hamiltonella* isolates were found in nature without the phage, but bottleneck passaging of the insects and symbionts generation after generation in the laboratory led to the loss of phage in multiple host lineages. Experimental assays confirmed that in the absence of phage, there was no protection [20]. Similarly, laboratory passaging of aphids and symbionts serendipitously led to spread of a mutation in the genome of *Buchnera aphidicola*, the primary, amino acid-synthesizing symbiont of pea aphids. The mutation, a single nucleotide deletion in the promoter for ibpA, a gene encoding for a heat-shock protein, lowers aphid fitness under elevated temperature conditions [21]. The mutation is found at low levels in natural aphid populations, suggesting that laboratory conditions facilitate maintenance of the genotype.

In the above cases, evolution was a fortunate coincidence. In this issue of *PLoS Biology*, Chrostek and Teixeira (2014) illustrate another alternative, directed experimental evolution. Previous work demonstrated that a strain of the symbiotic bacterium *Wolbachia*, wMelPop, is virulent to its *Drosophila melanogaster* hosts, considerably shortening lifespan while overproliferating inside the flies [22]. To investigate the mechanism of virulence, researchers compared the genomic content of an avirulent *Wolbachia* strain to that of the virulent wMelPop [12,13]. These comparisons revealed that the wMelPop genome contains a region with eight genes that is amplified multiple times; in avirulent strains there is only a single copy. This eight gene region was nicknamed “Octomom.” To functionally test whether Octomom mediates *Wolbachia* virulence, over successive generations, Chrostek and Teixeira selected for females with either high or low Octomom copy numbers to start the next generations. They found that copy number could evolve rapidly and was correlated with virulence. Flies harboring wMelPop with more copies of Octomom had shorter lifespans. This cost was reversed in the presence of natural enemies; flies harboring wMelPop with more copies of Octomom had higher resistance to viral pathogens. Thus, selection provided a functional link between genotype and phenotype in a symbiont recalcitrant to traditional microbial genetics approaches.

In many respects, this is similar to the research on aphids and their symbionts, where protective phenotypes were lost through passaging of aphids and symbionts generation after generation, as part of standard laboratory maintenance. Chrostek and Teixeira simply used the tools of experimental evolution to select for altered symbionts in a controlled fashion. Comparison of the studies also highlights two potential approaches—select for a phenotype and determine the genotypic change, or select for a genotype of interest and determine the phenotypic effect.

Why do we need to know the genetic mechanisms underlying symbiont-conferred traits? In terms of evolutionary dynamics, the maintenance of a symbiont’s effect in a population is predicated on the likelihood of it being maintained in the presence of mutation, drift, and selection. Symbiosis research often considers how ecological conditions influence symbiont-conferred traits but less often considers the instability of those influences due to evolutionary change. From the perspective of applied applications to human concerns, symbiont alteration of insect phenotypes are potential mechanisms to reduce vectoring of human and agricultural pathogens, either through directly reducing insect fitness or reducing the capacity of vectors to serve as pathogen reservoirs [23–28]. Short term field trials, for example, have demonstrated spread and persistence of *Wolbachia* in mosquito populations [29,30]. Because *Wolbachia* reduce persistence of viruses, including human pathogens, in insects [26,31–33], this is a promising pesticide-free and drug-free control strategy for insect-vectored diseases. Can we assume that *Wolbachia* and other symbionts will always confer the same phenotypes to their hosts? If the conferred phenotype is based on a region of the genome where mutation is likely (e.g., the homopolymeric track within the heat shock promoter of aphid *Buchnera*, the Octomom region in *Drosophila* wMelPop), then we have clear reason to suspect that the genotypic and phenotypic makeup of the symbiont population could change over time. We need to investigate how populations of bacterial symbionts evolve in host populations under natural ecological conditions, carefully screening for both changes in phenotype and changes in genotype over the course of such experimental observations. We then need to incorporate evolutionary changes when modeling symbiont maintenance and when considering the use of symbionts in applied applications.
